# Supplementary material for: What is the significance of a new breast mass in women aged 40 or above? A cross-sectional study
Source: PLoS One. 2026 Mar 20;21(3):e0341732. doi: 10.1371/journal.pone.0341732 (PMC13004385; doi:10.1371/journal.pone.0341732)
Supplement: Attachment — Submitted filename: Point by point Response.docx [file pone.0341732.s002.docx]

**Dear Dr. Giacomo Di Filippo,**

We sincerely appreciate the time and effort you and the reviewer have dedicated to evaluate our manuscript, "What Is the Significance of A New Breast Mass in Women Aged 40 or Above? A Cross-sectional Study" (Manuscript ID: PONE-D-25-37061). We are grateful for the constructive feedback, which has helped us improve the quality of our work.

We have carefully addressed all the comments and suggestions provided by the reviewer. Below, we provide a point-by-point response to each remark, detailing the revisions made in the revised manuscript. All changes have been highlighted in yellow.in the revised version for easy reference

**Journal Requirements:**

Comment 1*: “comply with PLOS ONE's requirements”*

Response 1: We ensured that the manuscript and files are formatted and named to meet all PLOS ONE style requirements.

Comment 2: *“We note that the grant information you provided in the ‘Funding Information’ and ‘Financial Disclosure’ sections do not match.”*

Response 2: Unfortunately we couldn’t find where to edit *‘Financial Disclosure’* in the submission portal. Please consider the following as our funding and financial disclosure:

*“This study was supported by a grant from the Deputy of Research of Tehran University of Medical Sciences, Tehran, Iran; Grant Number 1400-1-259-53034. The funders had no role in study design, data collection and analysis, decision to publish, or preparation of the manuscript.”*

Comment 3: *“Please state what role the funders took in the study.”*

Response 3: We added the necessary statement to our financial disclosure and cover letter.

Comment 4: *“Please note that funding information should not appear in any section or other areas of your manuscript.*

Response 4: We removed the funding information from our manuscript.

Comment 5: *“The data availability restrictions”*

Response 5: The data that support the findings of this article are not publicly available due to ethical concerns observed in the instructions of the Arash Registry of Breast Diseases Protocol. They can be requested from bdrc@tums.ac.ir.

Comment 6: *“The data contact point is a co-author, which violates policy; it must be a non-author institutional contact.”*

Response 6: According to this policy, Arash Registry of Breast Diseases’ contact address (bdrc@tums.ac.ir.) is presented as the data contact person. This has been corrected in the manuscript.

Comment 7: *“PLOS requires an ORCID iD for the corresponding author.”*

Response 7: The corresponding author linked his ORCID ID in the submission portal. His orcid number is: 0000-0001-7988-3050

Comment 8: *“Your ethics statement should only appear in the Methods section of your manuscript.”*

Response 8: The ethics statement now only appears in the Methods section of our manuscript.

**Editor:**

We sincerely thank you for your time and valuable feedback.

**Comment 1:** *“I believe the paper could use some refinement based on what the reviewer said. I also believe most of these issues are major in their nature.”*

**Response 1:** The manuscript has undergone a comprehensive and detailed revision to address all the major concerns raised by the reviewers.

**Reviewer #1:**

We are truly grateful for your kind and encouraging words, and sincerely thank you for your comment.

**Comment 1:** *"I only suggest revising in typos, punctuation, and double spaces as seen.”*

**Response 1:** The manuscript has been proofread (both by Grammarly and by an expert in English), and all typographical errors, punctuation, and extraneous spacing have been corrected.

**Reviewer #2**

Thank you for taking the time to review our work. We’re grateful for your time and valuable feedback.

**Comment 1:** *"Inconsistent Labeling of Protocols (P1 and P2)”*

**Response 1:** We apologize for the mistakes. We have re-written all related parts and renamed the protocols as “our institutional protocol” and “the guideline-based protocol” to prevent errors.

**Comment 2:** *"The "unwritten protocol" for biopsy decisions is vaguely described. For example, how was "new mass" defined (e.g., patient-reported vs. prior imaging comparison)? What thresholds were used for "suspicious clinical exam"?”*

**Response 2:** The relevant explanations have been added in the Methods section (lines 119-131).

**Comment 3:** *"Abbreviations like BE (breast exam), MG (mammography), and US (ultrasound) are not defined on first use.”*

**Response 3:** All abbreviations have been defined upon their first use in the revised manuscript.

**Comment 4:** *"Sample size justification is absent—was power calculated for detecting sensitivity differences?”*

**Response 4:** A sample size justification has been added (lines 133-151).

**Comment 5:** *"Please expand the methods to include a flow diagram of the protocol and define "new mass" explicitly (e.g., absent on prior imaging within 1 year).”*

Response 5: Thank you for the recommendation. The flow diagram has been added in the Methods section as Figure 1.

**Comment 6:** *"If applicable report inter-rater reliability for BI-RADS assignments if multiple radiologists were involved. This will enhance reproducibility.”*

**Response 6:** Unfortunately since the dataset does not identify the specific radiologists for each assignment, calculating inter-rater reliability is not possible. This limitation has been noted in the manuscript (lines 312-313).

**Comment 7:** *"Table 1 shows gravidity differences (p<0.001), but the text misinterprets; higher gravidity (≥5) is more common in malignant cases (34.81% vs. 14.56%), yet you state "fewer pregnancies" for benign—correct this.”*

**Response 7:** We made the necessary amendments (line 176).

**Comment 8:** *"No confidence intervals (CIs) for diagnostic metrics, limiting precision assessment. Please add 95% CIs to all metrics in Table 3.”*

**Response 8:** We added 95% CIs to all metrics in Table 3 (Table 3).

**Comment 9:** *"Please update to include 2024-2025 publications.”*

**Response 9:** This has been done, references from 2024 and 2025 have been used and included (Reference number: 12, 13, and 18).

**Sincerely,**

**Dr. Amirhossein Eskandari**
